# New Operation on the Foot

**Published:** 1851-08

**Authors:** 


### RECORD OF MEDICAL SCIENCE.

### SURGERY.

### MEDICAL SOCIETY OF LONDON, APRIL 5TH, 1851.

*New Operation on the Foot.*—Mr. T. Wakley read a paper on a new operation lately put in practice by him. It consists in dissecting out the astragalus and os calcis, and leaving the other tarsal and metatarsal bones undisturbed, and is intended in certain cases as a substitute for Syme's or Chopart's operations.

The author remarked that excision of joints was first attempted by Mr. Parkes, of Liverpool, in the year 1782, who had one successful case of excision of the knee-joint in a seafaring man, who was able to follow his calling nearly as well after the operation as before. Another patient operated on by Mr. Parkes died. Excision of the elbow-joint was performed three times in France in the same year with complete success: one of those operated upon was afterwards enabled to thrash wheat. Mr. Wakley then made some remarks on the process of anchylosis sometimes performed by nature in cases of diseased joints, and considered this an argument for the operation of excision; still, he remarked that the constitutional effect of this operation was more severe than that produced by amputation, and therefore it could not be performed with safety when the powers of the system were at a very low ebb. He considered the operation justifiable if the constitution be good, if performed in time, and with the necessary surgical skill. When the arm was operated upon our object should be to preserve the mobility of the elbow-joint, but in the lower extremity, when more firmness was requisite, anchylosis was the best result that could be obtained. The ankle-joint is generally considered as best adapted to the operation of excision, but Mr. Wakley in some cases preferred it to Chopart's or Syme's operation.

The author then remarked on the necessity of preserving, if possible, the anterior tibial and other arteries supplying the foot, so as to keep up its nutrition, and also upon the delicate dissection necessary to effect this object. Mr. Wakley then gave a short account of the case in which he had first performed this operation. The patient, a young man, of dark complexion and scrofulous habit, and whose occupation required a good deal of walking, had an open abscess just under the ankle-joint for two years; he then had the misfortune to sprain his ankle, and was admitted into St. George's Hospital: he was afterwards sent to Margate Hospital, when a piece of carious bone came away. He then became much better, and was enabled to return again to his work, but only for a month, after which period he was admitted into Brighton Hospital, where amputation at the ankle-joint was proposed; but to this operation the patient refused to submit, and he left accordingly. He was then admitted into the Royal Free Hospital, London, under the care of Mr. T. Wakley. On his admission, Dec. 21, 1847, the integument all around the heel and ankle-joint was much discolored and unhealthy in appearance, with three openings, two communicating with the astragalus, and one with the os calcis. On introducing a probe, rough bone could be detected; there was also a good deal of constitutional disturbance, and symptoms of hectic had appeared. Mr. T. Wakley, finding that the patient steadily opposed the removal of the foot, and having satisfied himself that the disease was confined to the os calcis and astragalus, proposed the removal of the two bones, to which the patient consented. The operation was performed on Dec. 27, 1847. The os calcis, astragalus, and the malleolar processes being removed, the posterior artery alone required tying; the wound healed kindly, and chiefly by the adhesive process: the man was then sent into the country, and soon regained his flesh, and is now in good health, with a tolerably useful limb, and without any appearance of the disease in any other part: both bones were so carious that they were mere shells.

Mr. Smith quite agreed that this had been a most successful operation, to which he had at first been opposed, as, although more than three years had elapsed, no reappearance of the disease had taken place.

Mr. B. Childs, who did not arrive until the paper was nearly concluded, did not consider Mr. T. Wakley's operation a new one; it was only a modification of a principle long recognized by surgeons, viz., that we should always in our operations spare as much as possible of the human frame: he considered the chief merit and difficulty of the case consisted in the diagnosis.

Mr. Clark remarked on the impropriety of a member making observations on a paper he had not heard, and also observed, that Mr. Wakley did not claim to himself the discovery of a new principle, but only an adaptation of the old-established one mentioned by Mr. B. Childs: he considered this operation might as fairly be called after the name of the inventor as those known by the names of Chopart's and Syme's operation were after their respective inventors.

Mr. E. Canton thought that in the paper sufficient attention had not been paid to diagnosis of whether these were the only bones diseased; thought Mr. Wakley fully entitled to give his name to the operation, though the point for inquiry was—Is this operation better than Syme's and why?

Mr. Gay said, that Mr. Wakley's patient could now go up a ladder, &c., and the tendo-achillis had so attached itself to the structure of the foot, that the gastrocnemius and solens had pretty complete control over its motions; thought that where the disease was limited and the constitution good, that this operation was sometimes preferable to Syme's; but as, in Mr. Wakley's operation, the convalescence was more tedious, and greater powers of reparation were necessary, thought Mr. Syme's best where there was much debility, although in the latter operation there was often a tendency to sloughing of the flap. Mr. Gay did not consider that a joint or bone in the neighborhood of another diseased bone was necessarily diseased also. He related a case in which, about three weeks ago, he had removed the os calcis, and found the surface of the astragalus quite denuded of cartilage; he did not, however, interfere with it, and although the youth was at the time of the operation in a very bad state of health, he is now rapidly recovering, with every prospect of a permanent cure.

Mr. Wakley in reply said, that he was not desirous that the operation should be called after him; had brought the case forward for the general good, and not for his own personal aggrandizement; thought this operation was only to be performed when the os calcis and astragalus were alone diseased. He could not agree with Mr. Gay, but thought that all the parts implicated by the disease should be removed.

*London Lancet.*

